# Participatory Design Approach in the Use of Scenario Analysis for Futureproofing Medical Education: Case Study

**DOI:** 10.2196/73173

**Published:** 2025-09-23

**Authors:** Guido Giunti, Ronan Glynn, Jack Hennessy, Colin P Doherty

**Affiliations:** 1 University of Oulu, Pentti Kaiteran katu 1, Oulu, 90570, Finland, 358 46921379; 2 St James's Hospital, Dublin, Ireland; 3 School of Medicine, Faculty of Health Sciences, Trinity College Dublin, Dublin, Ireland; 4 Ernst & Young (Ireland), Dublin, Ireland

**Keywords:** medical education, future studies, participatory design, health care professionals, megatrends

## Abstract

**Background:**

Medical education must evolve to prepare health care professionals for a rapidly changing world. Beyond digital literacy, clinicians must develop new competencies to navigate global megatrends, including shifting disease burden, technological advancements, climate change, and demographic shifts. The future job market will introduce novel roles, and educational institutions must remain adaptable to meet the evolving motivations and expectations of students. Megatrends, broad, transformative forces shaping societies, present both challenges and opportunities for health care education.

**Objective:**

The present work seeks to understand the implications of megatrends for medical education and explore the use of scenario analysis for curriculum design.

**Methods:**

A participatory design approach was employed to conduct a scenario analysis workshop at Trinity College Dublin’s School of Medicine in October 2024. Digital connectivity and climate change were selected as key drivers. Participants included medical educators, policymakers, clinicians, and students. Interactive methods such as group discussions, structured boards, and physical cards were utilized to facilitate data collection. Insights were analyzed thematically to identify critical competencies, mindsets, and structural requirements for future medical education.

**Results:**

The scenario analysis revealed key competencies and mindsets necessary for future health care professionals. Essential competencies included complex adaptive systems thinking, patient-centeredness, continuous learning, and participatory health, while essential mindsets encompassed sustainability, prevention-focused care, and technological adaptability. Cross-scenario reflections highlighted the increasing need for interdisciplinary collaboration, ethical leadership, and curriculum flexibility. Actionable steps were identified, including integrating sustainability and digital health into curricula, fostering emotional intelligence in student selection, and incorporating adaptive learning models.

**Conclusions:**

This study demonstrates the value of participatory design in shaping medical education to align with global megatrends. The findings align with existing foresight research by organizations such as the World Health Organization and the European Commission, emphasizing the need for health care professionals to balance technological proficiency with human-centered care. While the study was limited to a single institutional setting, its insights provide a framework for other medical schools to anticipate future challenges and proactively reform curricula. Future research should explore multi-institutional applications and longitudinal studies to validate these findings.

## Introduction

There is a clear need for health care professionals (HCPs) to become more acquainted with emerging technologies [1]; however, digital literacy is not the only skill that is needed. There has been a significant shift as countries have developed and the overall population has aged. Over 60% of all deaths are caused by chronic conditions like cancer or heart disease, taking precedence over acute infectious diseases, a so-called epidemiologic transition. Conditions that would previously have been rapidly disabling or fatal are now controllable if not curable. However, these changes are only part of the overall landscape: Migrations due to geopolitical instability and conflict and the search for better economic prospects and educational opportunities have resulted in clinicians facing unfamiliar diseases that were not common in their regional communities [2]. Climate change is impacting health through climatic extremes and multifaceted influences like regional changes in vector and pathogen distribution [3].

Medical schools in the 21st century face new challenges from the knowledge economy and must perform in a fierce global competition for talent [4,5]. The future job market is evolving, with new positions and roles continually emerging. The interest and motivations of younger generations to pursue higher learning are changing, leading to demands in curricula and employment opportunities to be able to accommodate and cater to a diverse array of motivations [6]. Health care education organizations need to remain relevant in a fast-changing world and balance stakeholder needs to ensure that students feel represented and valued [7], with many world-class universities adapting by developing new dynamic capabilities and changing their strategic governance [8-10].

Global transformative forces are called “megatrends” and define the future world with their far-reaching impact on businesses, societies, economies, cultures, and personal lives [11]. Megatrends are transformative, global forces that define the future world with their far-reaching impact on businesses, societies, economies, cultures, and personal lives [12]. These megatrends can interact with each other, creating complex and layered impacts on businesses and societies. The European Strategy and Policy Analysis System (ESPAS) “Global Trends to 2030” report [13] identifies certain megatrends including demographic shifts, technological innovation, and environmental challenges, which have direct implications for health care and education.

Futures studies is a generally accepted academic discipline to explore and formulate alternative futures [14,15]. There is a variety of methods to explore potential futures through futures studies [15,16]. Participatory design has emerged as an approach related to user-centered design to ensure that innovations meet the needs of their end users [17]. Participatory design involves end users directly in the design process, so that their voices shape the development of the tools they will eventually use. By promoting user-centered design, participatory design contributes to creating more effective, engaging, and acceptable solutions [18].

The present work seeks to understand the implications of megatrends for medical education.

## Methods

### Study Design

A participatory design approach was utilized to conduct a scenario analysis workshop in October 2024 to address Trinity College Dublin’s School of Medicine’s (TCD) strategic concerns. The qualitative approach was selected because of its effectiveness in offering insights into complex and multifaceted experiences, particularly when the primary aim of the study was to achieve a detailed and nuanced description [19,20].

Participatory design is an approach that actively raises the voices of all stakeholders and brings everyone to an equal level [21]. Scenario analysis is a process of evaluating and understanding possible future events by considering how key factors can lead to various feasible scenarios [16]. It is a method used in strategic planning to help anticipate uncertainties and develop strategies to address them.

Scenario analysis was used to generate visions of the future wherein medical education would need to take place, while participatory design was used as a means to engage participants in in-depth exploration of each future narrative and approach to co-creation of the required competencies and mindsets that might be needed in the future. TCD was used as an instrumental case; the focus of instrumental case studies is on gaining understanding of the phenomenon rather than on the case itself [22].

### Setting

TCD is one of the oldest medical schools in Europe and is in the process of upgrading its Medicine degree. TCD’s reform is facing the challenge of creating a program that would prepare HCPs for the world of tomorrow. TCD’s current strategic concerns are (1) ensuring curricula remain cutting-edge and globally relevant, (2) balancing the push toward digitization with hands-on clinical training, and (3) addressing the increasing importance of environmental and social responsibility in medical practice.

The objective was to develop an understanding of potential future scenarios for TCD and formulate strategies to address challenges and capitalize on opportunities for the Medicine degree program.

### Recruitment

An email invitation was sent to senior staff members at TCD, policy makers at the Health Service Executive, and selected medical students. Purposive sampling was used to ensure that the sample was rich for analysis. The sampling was based on several factors such as background, age, and years of experience, among other factors.

### Global Megatrends

The ESPAS “Global Trends to 2030” report [13] identifies the following megatrends:

- Climate change is perhaps the most pressing global challenge, necessitating a strategic and adaptive response from all sectors. The health care industry, as a significant contributor to global greenhouse emissions, faces a dual role in both mitigating its environmental impact and preparing for the health implications of a changing climate.
- Technological innovation continues to advance at a rapid pace and is poised to significantly enhance health care delivery and efficiency. Artificial intelligence (AI) and robotics are at the forefront of this advancement and offer the potential for improved patient outcomes and streamlined operations, yet also introduce complex challenges, including the displacement of job functions, ethical dilemmas, and heightened cybersecurity risks.
- Demographic shifts are reshaping global population distribution, with significant growth in regions like Sub-Saharan Africa and South Asia, contrasted by an aging population in Europe. In Ireland, notable recent population growth has been driven by positive net migration, underscoring the broader trend of migration as a key factor in demographic changes and the need for sustainable resource management and health care adaptation.
- Economic patterns indicate a steady rate of global growth, with the expansion of the middle class and a reduction in extreme poverty, highlighting the strides made in economic development. However, wealth distribution remains uneven, with a small percentage of the population expected to hold a disproportionate share of global wealth.

### Scenario Generation

Deliberations took place in the form of structured group discussions with TCD’s executive leadership team in order to determine the most relevant drivers for TCD, reflecting the most pressing concerns and anticipated external pressures relevant to future curriculum design. The aim was to align scenario construction with institutional foresight rather than explore low-probability, high-uncertainty events. After deliberation, *Digital Connectivity* and *Climate Change* were identified as the two key drivers for this session as these factors fundamentally influence technological adoption, health care delivery models, and sustainability challenges. Once these drivers were selected, the team developed a scenario matrix, mapping out four distinct future scenarios based on high and low variations of each driver.

To bring these scenarios to life, the team employed ChatGPT for narrative generation, using detailed prompts to craft scenario descriptions that were specific to the strategic needs of the study. The narratives were designed to immerse participants in each possible future, emphasizing the elements and contexts relevant to the discussion. To create further engagement with the scenarios, MidJourney was used to create compelling visual representations, ensuring that the abstract concepts were visually evocative and memorable. Finally, the narratives were transcribed using Murf.AI, an AI voice generator, and played during the workshop sessions to create an immersive experience that helped participants conceptualize the future as a lived reality.

The use of AI was deliberate to explore how these tools might support scenario planning. All outputs were reviewed and refined by human facilitators. While each scenario presents distinct challenges and opportunities, overly dystopian or utopian framings were avoided to ensure balance.

### Data Collection

The event was held in a dedicated off-site innovation space, such that participants could have the opportunity to break free from the everyday, to gain a fresh mindset and to use collaborative tools to reframe their thinking.

The session employed various interactive methods, including presentations, group discussions, and hands-on activities, to foster an environment of collaboration and shared learning. Participants were presented with information from the ESPAS report, were introduced to the scenario analysis methodology, and were then presented with four plausible future scenarios of what the world and Ireland would look like in the year 2035.

An ad hoc set of materials was designed to facilitate data collection and effectively guide participants through the participatory design workshop. Recognizing that scenario analysis can be complex and unfamiliar to many, we developed a structured board that visually organized the process, breaking it down into clear, digestible steps. This served as a focal point for participants, ensuring they could navigate the method intuitively. In alignment with embodied cognition theory [23,24], physical cards were created to make abstract concepts more tangible. These cards represented essential future mindsets and skills for HCPs, derived from relevant literature [25-29]. Blank cards or wildcards were also included to allow participants to introduce new mindsets and skills that were not represented in the provided cards.

Additionally, we prepared printout slides containing the different scenario descriptions, providing key details such as the overarching narrative, global context, and defining features of each scenario.

Participants were divided into 4 groups of up to 7 people, each focusing on a separate scenario. Each group participated in timed sessions that transitioned from passive immersion to active engagement with the scenarios.

Throughout the session, data was systematically captured through notes, photographs, and sticky notes, ensuring that discussions, insights, and decision-making processes were well-documented for later analysis.

### Data Analysis

A group sorting approach combined with thematic analysis was utilized to systematically examine the insights generated during the workshop. The analysis incorporated multiple data sources (sticky notes, facilitator notes, photographs, notes from participants’ presentations). Thematic analysis was conducted in an iterative and inductive manner [30], allowing key concepts and patterns to emerge organically from participant contributions rather than being imposed by predefined frameworks.

The collected materials were categorized and sorted into broad thematic clusters, focusing on competencies, mindsets, and structural needs for future health care education. The discussions and presentation notes were transcribed and analyzed for recurring themes, divergences, and underlying assumptions. In order to help triangulate findings across different data points, structured outputs from scenario discussions and the more fluid, reflective insights from open conversations were integrated. The approach allowed for the capture of consensus areas and areas of debate and uncertainty. Triangulation was achieved by cross-referencing themes that emerged across the multiple formats (eg, sticky notes, verbal group summaries, facilitator observations), allowing validation of recurring patterns and identification of unique contributions across the different data types. Shenton’s guidelines were followed [31] to help ensure the integrity of the content analyses and generate an audit trail.

### Researcher Reflexivity

Researcher reflexivity was critical throughout the case: the lead author is a digital health expert with training in applied strategic foresight and ample experience in participatory design, and the facilitators brought expertise as HCPs and experienced strategy consultants. This positionality shaped both the design and execution of the workshop, influencing how scenarios were framed, how discussions were guided, and how emerging insights were interpreted. Our backgrounds allowed us to provide depth in discussions on digital transformation, health care systems, and future competencies but also required constant self-awareness to mitigate bias, ensuring that we did not unintentionally steer conversations toward predefined narratives or overlook alternative perspectives. To foster participant-driven insights, we emphasized an open-ended, exploratory approach, where diverse voices were prioritized and deliberation was structured around materials that encouraged co-creation rather than facilitator-led direction. The facilitators adopted an observational stance during the workshop sessions to minimize bias in participant interaction. By reflecting on our roles throughout the process, we remained mindful of how our expertise might shape participant interactions and interpretations, striving to balance guidance with openness, allowing emergent themes to be driven by the collective experience of the group rather than our own professional frameworks.

### Ethical Considerations

The work was carried out in accordance with the ethical principles of research with human participants and ethical review in the human sciences in Finland [32]. The ethical review process was not required for the present study as it involved participants in their capacity as experts. The activities conducted were designed for knowledge sharing within a professional setting, participants were informed about the purpose and objectives of the session and consented to participate.

## Results

### Scenarios

#### Overview

**Figure 1.**
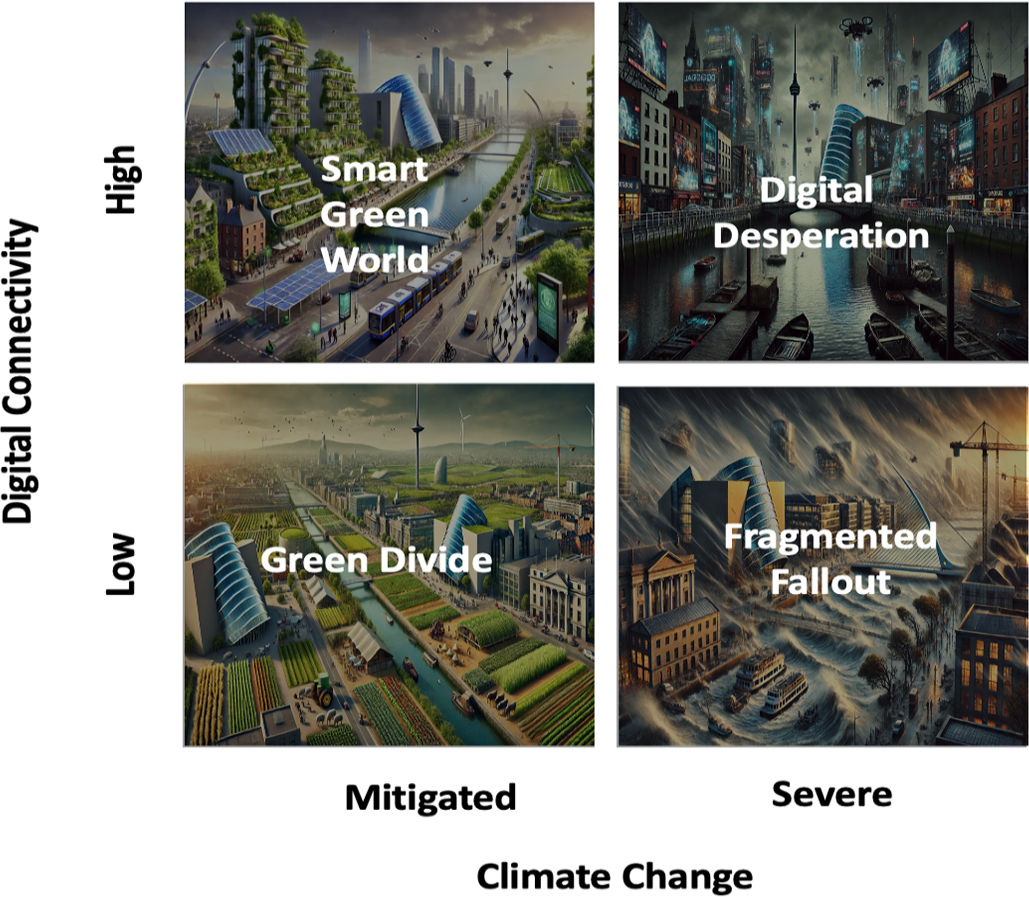
Graphical representations of each scenario, arranged in a scenario matrix.

The following scenarios reflect divergent futures based on the chosen drivers. Table 1 shows the resulting scenario matrix using Digital Connectivity and Climate Change, while Figure 1 provides graphical representations. A brief description of the different scenarios and their respective narratives is described below for illustrative purposes.

**Table 1.** Scenario matrix using digital connectivity and climate change overview.

Scenario	Digital connectivity	Climate response	Health care context	Key risks	Key opportunities
Smart Green World	High	Effective	Preventive, AI-led^a^	Data privacy, tech access	Sustainable care, equity
Digital Desperation	High	Poor	Stratified access	Inequality, misinformation	Tech innovation, resilience
Green Divide	Low	Effective	Localized care	Regional inequity	Community engagement
Fragmented Fallout	Low	Poor	System collapse	Migration, instability	Grassroots innovation

a AI: artificial intelligence.

#### Smart Green World

In this scenario, high digital connectivity and successful climate change mitigation lead to a globally interconnected and sustainable society. International cooperation thrives, powered by green technologies and digital platforms. Health care relies heavily on AI-driven diagnostics and telemedicine, with strong emphasis on preventative care. Challenges around data privacy and equitable access to smart technologies persist.

#### Digital Desperation

While digital connectivity is high, climate change accelerates without meaningful mitigation efforts, creating stark inequality. Advanced technologies and health care innovations are available to the wealthy, while the majority of the population struggles with climate disasters, resource scarcity, and inadequate health care. There is growing social unrest and health crises in under-resourced areas. Misinformation spreads quickly, exacerbating social tensions.

#### Fragmented Fallout

In this bleak scenario, both global connectivity and climate change mitigation have failed, leading to widespread social and economic collapse. Climate disasters are rampant, international trade and cooperation are hampered, and health care systems are overwhelmed by shortages of resources. Mass migration, conflict over dwindling resources, and extreme poverty characterize the global landscape.

#### Green Divide

Climate change is mitigated, but global connectivity weakens, resulting in regions becoming more self-sufficient. While wealthier areas leverage renewable energy and green technologies to thrive, poorer regions struggle to keep pace. Health care systems focus on local needs, with cities benefiting from telemedicine and advanced diagnostics, while rural areas face limited access to medical technology. Inequality drives political instability, and regions that have not benefited from green technologies face risks from climate change.

### Participatory Workshop

The event gathered 28 participants ranging from medical students, HCPs, academicians, and policy makers. Table 2 presents some descriptive characteristics of the participants.

**Table 2.** Participant characteristics.

Characteristics	Description
Gender	There was an even distribution (53% male), with representatives of both genders present.
Age	The age of participants ranged from early 20s to late 50s, with a relatively wide age distribution, with a mix of early-career and senior professionals.
Background	Participants included a majority of HCPs^a^ (39%, n=11), followed by policy makers (25%, n=7), academics (22%, n=5), and medical students (14%, n=4). This variety highlights a multidisciplinary approach to health care within the sample.
Years of experience	While some health care professionals, academics, and policymakers had extensive backgrounds in their respective fields, others, such as students, were at the early stages of their medical careers. This variation in experience contributed to a rich and multifaceted discussion.

a HCP: health care professional.

**Figure 2.**
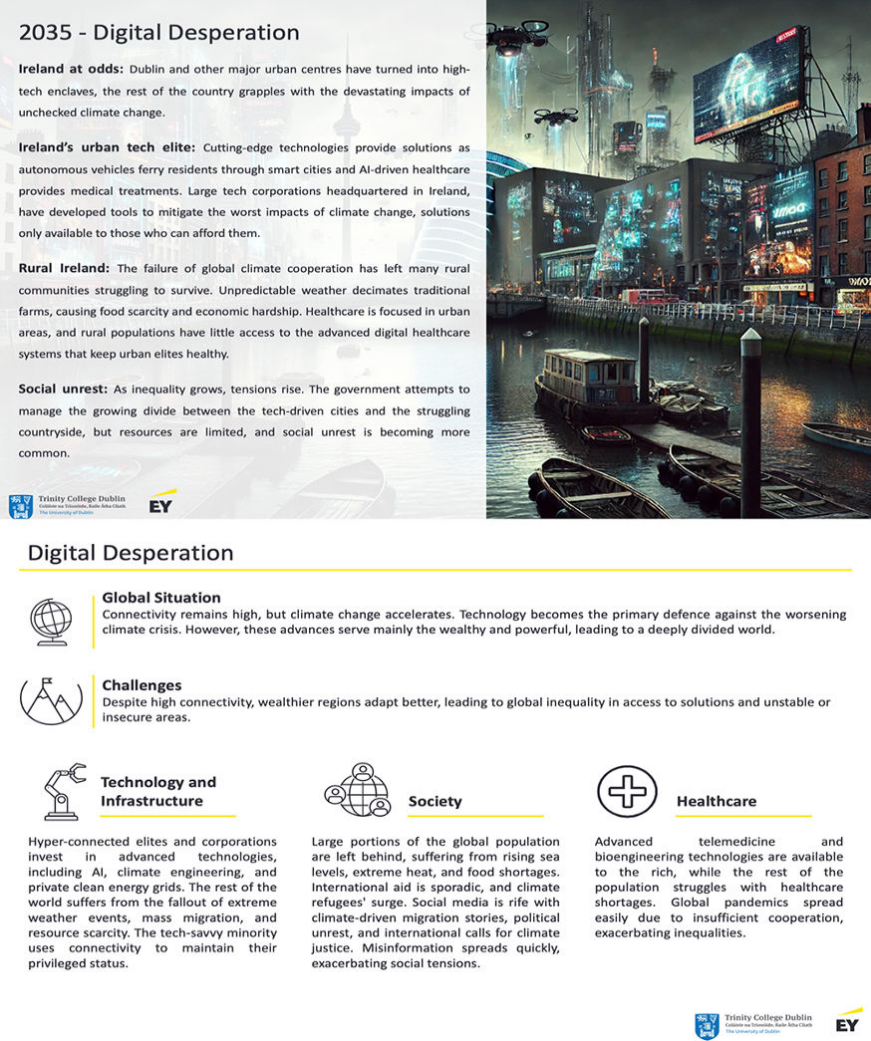
Sample printout of a scenario. Top: Front of the printout with the scenario’s narrative. Bottom: Back of the printout with highlights for the participants.

**Figure 3.**
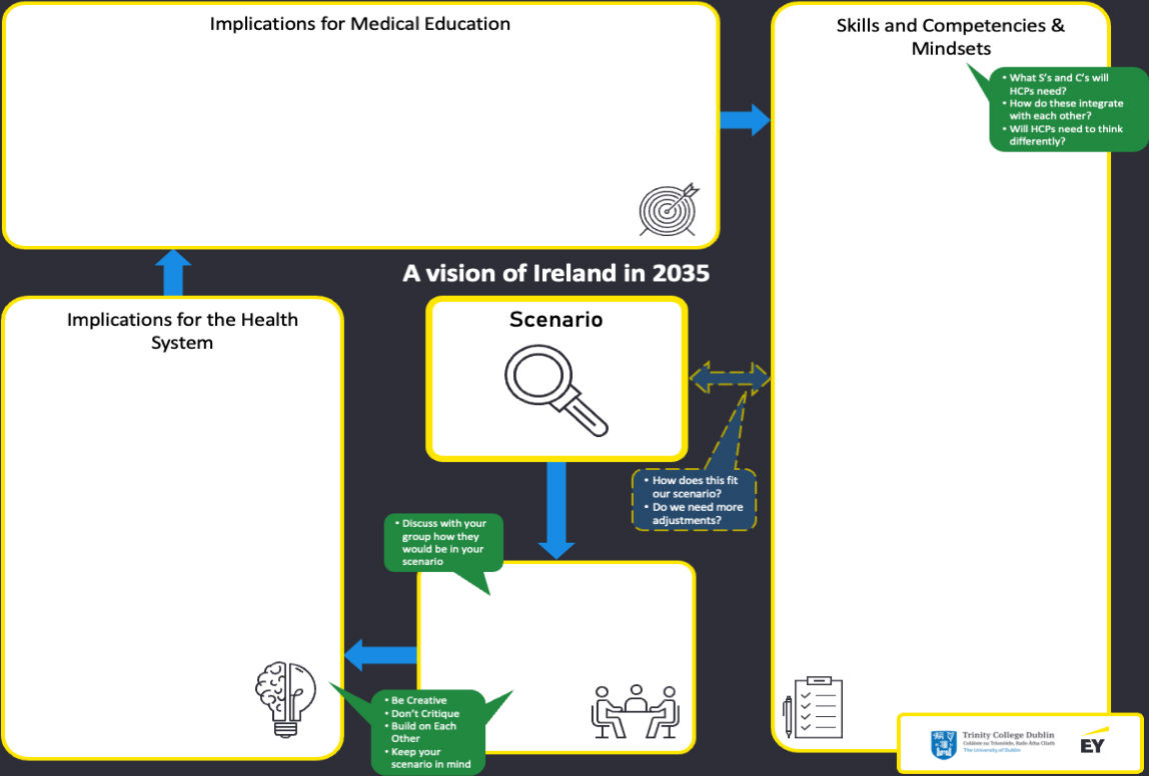
Structured board representing steps in the workshop with the scenario placed at the center.

**Figure 4.**
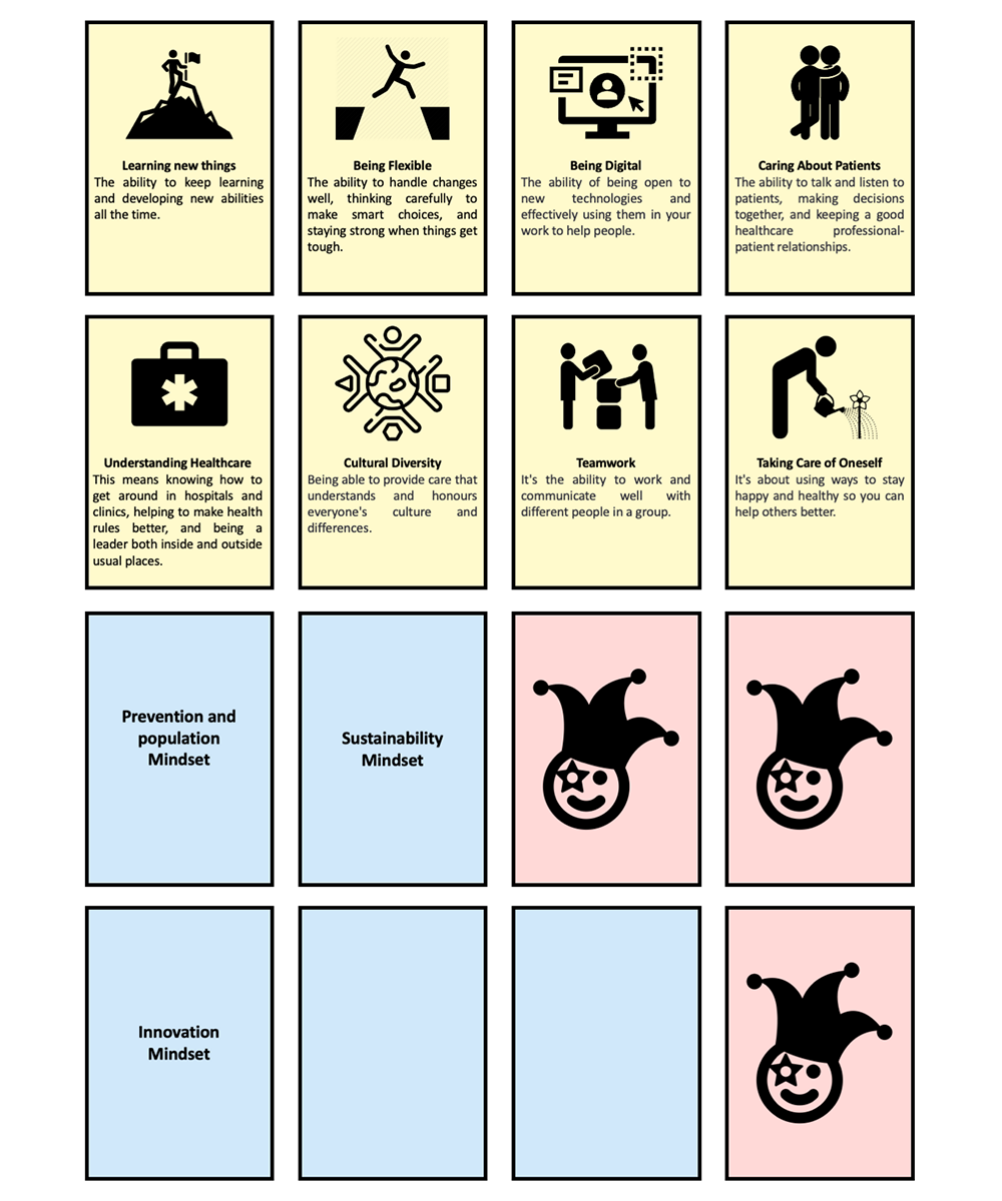
Physical cards representing competencies (yellow), mindsets (blue), and wildcards (pink).

Participants were divided into groups of 7 individuals, ensuring that there would be a mixed variety of backgrounds in each group. Each group received a printout of the scenario (Figure 2) and was assigned a structured board (Figure 3). The groups moved through timed segments from discussing the scenario and its implications for common people to more specific health care issues and needs for medical education. Sets of physical cards containing competencies and mindsets were provided to trigger conversation (Figure 4), as well as wildcard cards that could be used to represent unknown or unaccounted elements.

The activity concluded by outlining strategic training actions that medical schools would need to take now to be ready for that future. At the end of the session, all groups presented their work, and the floor was opened for free exchange.

### Cross-Scenario Reflections

Across all four scenarios, the competencies and mindsets were selected as essential for HCPs of the future (Textbox 1).

Textbox 1. **Competencies and mindsets**

**Competencies**

- Complex adaptive system thinking: Professionals will need to understand and operate within highly complex health care systems that are influenced by technology, climate, and societal changes.
- Patient-centeredness: Despite the rise of digital tools, maintaining a human-centered approach in patient care will remain crucial.
- Continuous learning: The ability to learn new skills and adapt to technological changes will be vital as health care systems evolve.
- Participatory health: Encouraging patient participation in health decisions, supported by digital tools, will become a core aspect of future health care delivery.

**Mindsets**

- Eco-friendly, sustainability mindset: A focus on environmentally friendly health care practices, minimizing carbon footprints, and promoting public health will be key.
- Prevention and population health: A shift toward preventive care and addressing health at a population level rather than just individual treatments.

- Technology and personalized medicine: Training health care professionals to embrace new technologies and tailor medical care to individual needs through AI and data-driven tools.

Furthermore, the following emerging aspects were identified during group work and open discussions:

- Social responsibility: Emphasis on ethical leadership and a focus on the human connections in healthcare.
- Rethink selection criteria for students: Selecting individuals who demonstrate both technical and emotional intelligence will be critical.

### Actionable Steps

There was a clear interest and motivation in improving medical education with a strong desire to be involved in the process. The session highlighted the need for a long-term vision and strategic planning; however, there were actionable steps that could be further defined to create short-term to medium-term actions. These are presented in Textbox 2.

Textbox 2. **Actionable steps for the future of medical education.**

Introduce complex adaptive systems thinking early through foundational courses on health care systems, public health, and the social determinants of health. Utilize real-world challenges involving multiple stakeholders, technologies, and societal factors with other faculties.

Embed patient-centered care in clinical training with communication skills, ethics, and empathy exercises woven into all clinical rotations. Develop assessments that measure these interpersonal skills in patient interactions.

Build flexibility into the curriculum with self-directed learning modules and electives in emerging technologies, AI, or digital health. Include reflection assignments that encourage students to identify areas for personal development and future learning.

Offer specific modules on participatory health, teaching students how to engage patients in decision-making using digital tools. Provide practical workshops on the use of health apps, wearable technology, and telemedicine in patient care.

Provide dedicated sessions on environmental sustainability in health care early, covering topics like green health care practices, the impact of health care on climate, and strategies for reducing carbon footprints in hospitals. Encourage students to explore sustainability through research projects.

Integrate leadership training and ethics discussions into modules. Require students to take on leadership roles in community initiatives to foster these skills. Incorporate preventive care into assessments, requiring students to devise prevention plans for patients.

Implement new selection processes that assess both emotional intelligence and technical aptitude. Use scenario-based interviews, emotional intelligence testing, or multiple mini-interviews to evaluate empathy, teamwork, and ethical decision-making. Continuously assess along academic performance.

## Discussion

### Principal Findings

This study makes contributions to the field of medical education and strategic foresight by employing a participatory design approach to explore the implications of global megatrends on the future of medical training. Through scenario analysis, it provides a structured framework for envisioning potential futures and identifying the key competencies and mindsets essential for future HCPs. By integrating stakeholder engagement, including policymakers, educators, clinicians, and students, the study ensures that its findings are grounded in diverse perspectives. Moreover, it offers actionable strategies for medical education reform, emphasizing adaptive learning models, sustainability integration, digital health training, and patient-centered approaches. By advancing a future-oriented, systems-thinking approach, this work equips medical schools with the tools to proactively navigate uncertainty and drive curriculum innovation in response to an evolving health care landscape. This study also offers a novel application of AI tools (eg, ChatGPT, MidJourney, Murf.AI) in foresight-driven educational planning.

### Comparison With Prior Work

The use of future studies to understand the implications for health care has been adopted by institutions such as the World Health Organization [33], the European Commission [13], and the United Kingdom’s National Health Service [16]. Scenarios, as part of strategic analysis, allow organizations to envision multiple futures, preparing them for a range of possibilities rather than a fixed trajectory [34]. They are useful in a rapidly changing world, enabling proactive rather than reactive strategies. Scenarios are widely acknowledged as an effective tool for enabling communities of practice to generate critical insights into the future and have been used in the past to prepare health care systems like National Health Service in the United Kingdom [16].

While the scenarios explored qualitatively different futures, many of the competencies and mindsets selected by the participant groups converged, suggesting that certain capabilities are seen as universally necessary. However, nuances in action prioritization did emerge across scenarios (eg, scenarios with low connectivity emphasized community-based solutions, while high-tech scenarios emphasized digital adaptability).

Participatory design has been used in health care in many instances for creating digital health solutions and educational content and experiences [17,18,21,35-37] and is generally aligned with the vision for public and patient involvement [38-40]. The approach allows stakeholders to have a voice in the systems, services, or processes that are relevant to them, advocating for a combination of backgrounds and experiences. Bringing in HCPs, policy makers, academicians, and medical students provided an opportunity for them to express their views. However, studies bring to light how power dynamics in participatory sessions may influence mixed group interactions [41-43]. Utilizing physical elements like cards to represent abstract concepts is an established practice in education in alignment with embodied cognition theory [23,24].

The findings from this study are aligned with overall concerns regarding medical education as the health care sector navigates through the complexities of the 21st century [44]: the need for enabling cooperation between HCPs and AI-enabled tools [29,45], promotion of empathy and personal resilience [46], and understanding of the impact of climate change and its implications on health care [47].

The application of AI tools to facilitate rapid development and visualization has been gaining traction in recent years [48,49]; however, this approach has raised concerns and questions around transparency, authorship, and potential bias issues [49-51].

The findings from this scenario-based participatory process inform the ongoing curriculum redesign discussions at TCD. These findings can help contribute to theoretical models of competency-based medical education by providing examples of how foresight methodologies can uncover both core and context-contingent educational needs. Furthermore, the study supports the emerging field of educational future literacy, which advocates for anticipatory capacity building in higher education institutions.

There is also interest in replicating this methodology at other institutions within and beyond Ireland to further validate its utility and adaptability. Finally, the study raises an important scientific and pedagogical question: How can medical education systems build internal foresight capacity, not just to anticipate future needs but to adapt in real time? This shifts the conversation from “what future to prepare for” to “how to maintain curriculum resilience in the face of uncertainty,” a shift that aligns with complexity theory and systems-based approaches to organizational learning.

### Limitations

This study employed qualitative research methods to explore future competencies and mindsets for medical education. While this approach provides rich, contextual insights, several limitations must be acknowledged.

The driver selection process, while institutionally relevant, did not follow a formalized Social, Technological, Economic, Environmental, and Political (STEEP) process, which may have introduced framing effects or excluded other impactful variables (eg, political instability). The findings emerged from a single workshop with 28 participants, representing a specific institutional and geographic context (TCD in Dublin). While key themes align with broader global trends in medical education, findings are not universally generalizable. A larger and more diverse participant pool, cross-institutional studies, and longitudinal follow-up research would help validate and refine these insights.

The background of the research team may carry implicit biases in scenario selection, discussion facilitation, and data interpretation. While open-ended prompts and participant-led discussions were designed to mitigate undue influence, facilitator expertise may have subtly shaped the direction of the conversations.

As the groups were mixed, the workshop environment inherently carries risks of groupthink and social desirability bias, where participants may align their views with dominant perspectives or the opinions of more vocal group members. No patient representatives were present in the session, and medical students’ voices could have been influenced by unexplored power dynamics. Efforts to counteract this included structured activities and diversified discussion formats, but the possibility of power imbalances or self-censorship remains.

The analysis relied on thematic coding of qualitative data from sticky notes, discussions, and participant presentations. Qualitative data is subject to researcher interpretation, making findings inherently subjective. The selection of key scenario drivers was expert-driven, based on foresight methodologies. While these drivers are highly relevant to the future of health care, alternative drivers could have led to different scenario framings and insights. Despite these limitations, this study provides valuable foresight into the future of medical education and demonstrates the utility of participatory methods in shaping strategic curriculum design.

Due to the nature of the workshop design, participant contributions were captured primarily via sticky notes, presentation summaries, and facilitator notes. While this allowed for triangulation of themes, the absence of direct transcriptions limits the depth of participant voice that can be directly quoted.

## Abbreviations

AI: artificial intelligence
ESPAS: European Strategy and Policy Analysis System
HCP: health care professional
STEEP: Social, Technological, Economic, Environmental, and Political
TCD: Trinity College Dublin’s School of Medicine
